# Role of Rho GTPases in inflammatory bowel disease

**DOI:** 10.1038/s41420-023-01329-w

**Published:** 2023-01-23

**Authors:** Xiaoling Li, Mudan Zhang, Gaoshi Zhou, Zhuo Xie, Ying Wang, Jing Han, Li Li, Qirui Wu, Shenghong Zhang

**Affiliations:** Division of Gastroenterology, The First Affiliated Hospital, Sun Yat-sen University, Guangzhou, PR China

**Keywords:** Inflammatory bowel disease, Molecular biology

## Abstract

Rat sarcoma virus homolog (Rho) guanosine triphosphatases (GTPases) function as “molecular switch” in cellular signaling regulation processes and are associated with the pathogenesis of inflammatory bowel disease (IBD). This chronic intestinal tract inflammation primarily encompasses two diseases: Crohn’s disease and ulcerative colitis. The pathogenesis of IBD is complex and considered to include four main factors and their interactions: genetics, intestinal microbiota, immune system, and environment. Recently, several novel pathogenic components have been identified. In addition, potential therapies for IBD targeting Rho GTPases have emerged and proven to be clinically effective. This review mainly focuses on Rho GTPases and their possible mechanisms in IBD pathogenesis. The therapeutic possibility of Rho GTPases is also discussed.

## Facts

- Rho GTPases are vital in numerous cellular events and associated with IBD pathogenesis.
- Rho GTPases participate in traditionally believed pathogenic factors of IBD.
- Roles of Rho GTPases in several novel pathogenic factors of IBD are also proposed.

## Open Questions

- Exactly what roles Rho GTPases play in IBD pathogenesis and what are the specific mechanisms?
- How to optimize the experimental design to explore the connections between Rho GTPases and IBD?
- Can we invent more secure and effective methods for IBD treatment by targeting Rho GTPases?

## Introduction

Characterized by progressive and chronic relapsing intestinal inflammation, inflammatory bowel disease (IBD) is likely to occur early in life and is unfortunately incurable [1]. Despite having an unclear pathogenesis, studies have indicated the involvement of genetic variants, environmental changes, abnormal intestinal microbiota, and immune response dysregulation, and the interactions between these factors ultimately leads to the onset of IBD [2]. In recent years, many novel pathogenic factors have been identified that provide new insights into the disease [2].

**Fig. 1:**
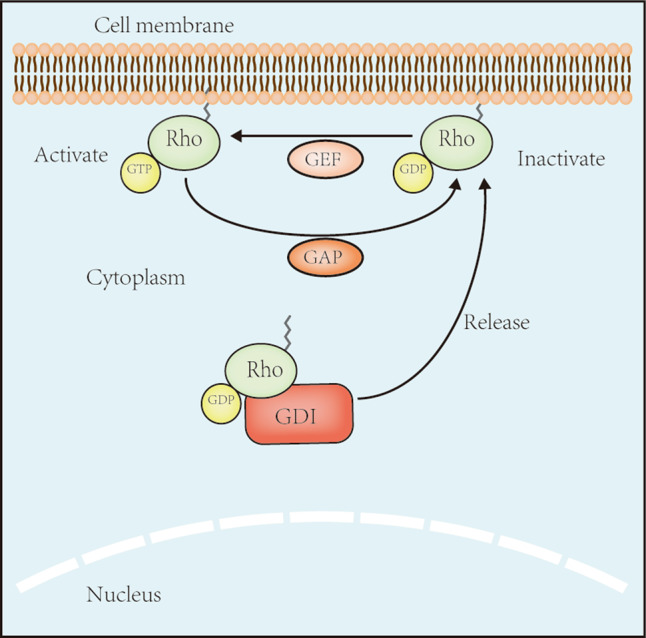
Schematic diagram of Rho GTPases molecular switches and its regulators. Rho GTPases are members of the Ras superfamily, acting as molecular switches and consisting of inactive GDP-bound and active GTP-bound forms. In the inactive GDP-bound form, GEFs promote the activation of Rho GTPases by replacing GDP with GTP, thus Rho GTPases change its conformation and can bind and activate various downstream effectors in order to trigger multiple cellular functions. By contrast, GAPs increase the hydrolysis of GTP to GDP and inhibit the activation of Rho GTPases. GDIs interacts with the GDP-bound Rho GTPases, preventing its localization to the cell membrane and maintaining Rho GTPases in the inactive form. When GDIs released, GDP-bound Rho GTPases will anchor to the cell membrane and be further activated by binding to GEFs. GEFs guanine nucleotide exchange factors, GAPs GTPase-activating proteins, GDIs guanine nucleotide dissociation inhibitors.

Rat sarcoma virus (Ras) homolog (Rho) guanosine triphosphatases (GTPases), a subgroup of the Ras superfamily, are guanosine triphosphate (GTP)-bound proteins [3]. The regulation of Rho GTPase activity depends on guanine nucleotide exchange factors (GEFs), GTPase-activating proteins (GAPs), and guanine nucleotide dissociation inhibitors (GDIs). GEFs enhance the switch of Rho GTPases from guanosine diphosphate (GDP)-bound forms to GTP-bound forms to activate them, whereas GAPs and GDIs inactivate them by increasing bound GTP hydrolysis and binding directly to them, respectively [4] (Fig. 1). Rho GTPases are vital in the cytoskeleton regulation and gene expression [3, 5]. Recently, studies have found that Rho GTPases are associated with numerous cellular events, therefore being important in chronic diseases such as IBD. Within the Rho GTPase family, cell division cycle 42 (Cdc42), Ras-related C3 botulinum toxin substrate 1/2 (Rac1/2), and Rho family member A (RhoA) are the most thoroughly studied proteins.

In this review, the functions of Rho GTPases in the pathogenesis of IBD and their possible mechanisms are summarized. The therapeutic strategies associated with Rho GTPases that may be beneficial to IBD patients and their clinical applied prospect are also discussed.

## Genetics

In recent years, over 200 loci have been found to be implicated in IBD. Among these loci, most increase the risk of both IBD subtypes, whereas several are unique to either CD or UC [6]. Rho GTPases are involved in this pathogenic process (Table 1).

**Table 1:** Effects of Rho GTPases-associated genetic variants in IBD.

Rho GTPases	Gene	variant	Disease	Model	Effect	Reference
Rac2	Rac2	rs6572 SNP	CD	CD patients	Rac2 SNP rs6572 is associated with CD.	[8]
		rs2899284 SNP	CD	CD patients	Rac2 SNP rs2899284 is associated with CD.	[9]
	NCF2	Heterozygote variant c.113 G → A	IBD	VEO-IBD patients	The NCF2 c.113 G → A variant links to VEO-IBD. This variant disrupts its product p67phox binding to RAC2 and then the formation of NADPH complex.	[8]
Rac1	Rac1	rs10951082 SNP rs4720672 SNP	UC	UC patients Mice with conditional Rac1 knockout	Rac1 SNPs rs10951082 and rs4720672 function in the susceptibility to UC. The risk alleles result in higher level of Rac1, which enhances neutrophil accumulation and the expression of pro-inflammatory cytokines in the colon.	[10]
		rs34932801 SNP	CD	Adult CD patients	Rac1 SNP rs34932801 result in CD patients poorer response to thiopurines therapy.	[11]
	ATG16L1	T300A SNP	CD	Human DCs Murine DCs	ATG16L1 SNP T300A is associated with CD. Decreased level of ATG16L1 protein improves Rac1 activity and then alter DCs migration.	[19]
Rho	RhoA Pggt1b	/	IBD	Intestinal tissues of IBD patients, Mice with conditional loss of RhoA/Pggt1b	Decreased expression of Pggt1b and impaired RhoA signaling are discovered in IBD patients. Reduced expression of Pgg1b in IECs disrupts RhoA membrane localization and activation, which causes damaged gut barrier, accumulation of immune cells in gut and upregulated pro-inflammatory cytokines level.	[13, 18]
	Myo9B	rs962917 SNP rs1545620 SNP rs2305764 SNP	IBD	IBD patients Caco2BBe (BBe) cells HeLa cells	Myo9B SNPs rs962917, rs1545620 and rs2305764 contribute to IBD. Dysfunction of Myo9B leads to increased Rho signaling and then the impaired IECs migration and localization of TJ proteins.	[14–16]
	ATG16L1	T300A SNP	CD	HT29 cells Human colonic tissue	ATG16L1 SNP T300A is associated with CD. Decreased level of ATG16L1 protein downregulates RhoA activity and then interrupts epithelial cell migration.	[20]
	TTC7A	Biallelic missense mutations	IBD	Patients with immune deficiency–related ELA syndrome	Biallelic missense mutations of TTC7A are related with early-onset IBD. The resulting reduction of TTC7A protein enhances RhoA signaling and then damages IEC polarity and disrupts T cell cytoskeletal remodeling.	[21]
	TAGAP	rs212388 SNP	CD	CD patients	TAGAP SNP rs212388 is negatively associated with the severity of anal disease of CD patients. The association might be due to TAGAP allele results in promoted Rho activity and then enhanced T cell-induced immune defense.	[22]

*CD* Crohn’s Disease, *NCF2* neutrophil cytosolic factor 2, *IBD* Inflammatory Bowel Disease, *UC* Ulcerative Colitis, *SNP* single nucleotide polymorphism, *VEO-IBD* very early onset IBD, *ATG16L1* autophagy related protein 16 like protein 1, *DCs* dendritic cells, *MYO9B* myosin IXB, *IECs* intestinal epithelial cells, *TJ* tight junction, *TTC7A* tetratricopeptide repeat domain 7A, *ELA syndrome* enteropathy-lymphocytopenia-alopecia syndrome, *TAGAP* T-cell activation Rho GTPase activating protein.

### Genes encoding Rho GTPases

Rac2 deficiency is associated with worsening colitis in mice treated with *Citrobacter rodentium*, implying a possible effect of this protein on human intestinal diseases development [7]. This hypothesis is supported by the fact that several *Rac2* single nucleotide polymorphisms (SNPs) may function in the predisposition and susceptibility to CD [8, 9]. Interestingly, whether the association between Rac2 and IBD is based on the function of Rac2 in the NADPH complex is controversial [7, 8]. The *Rac1* SNPs rs10951982 and rs4720672 are associated with UC [10], and another *Rac1* SNP, rs34932801, was found to lead to poorer thiopurine treatment effect in adult CD patients [11]; however, the association between *Rac1* SNPs and UC was not observed in another study [12]. In addition, mice with a genetic deletion of *RhoA* in intestinal epithelial cells (IECs) exhibited spontaneous chronic intestinal inflammation, and RhoA signaling was found to be downregulated in CD patients [13].

### Other genes associated with Rho GTPases

#### Myo9B

Genetic variations in myosin IXB (*Myo9B*), a Rho GAP encoding gene, are associated with IBD [14, 15]. This association might be partly explained by the dysfunction of Myo9B, leading to an abnormal level of active Rho, subsequently influencing intestinal epithelial wound healing and disrupting the formation of tight junctions (TJs) [16].

#### Pggt1b

Protein geranylgeranyltransferase type-I (GGTase-I) transfers a 20-carbon geranylgeranyl lipid to substrate proteins, including Rho GTPases [17]. This process is a post-translational modification of Rho GTPases called prenylation. Genetic deletion of *Pggt1b*, a gene encoding GGTase-I, in murine IECs results in epithelial injuries and spontaneous gut inflammation, which can be significantly ameliorated by activating RhoA signaling [13]. Loss of *Pggt1b* in the T cells of mice also causes spontaneous colitis via impaired RhoA signaling, and the intestinal T cells of IBD patients have lower *Pggt1b* level [18].

#### ATG16L1

The SNP T300 of autophagy-related protein 16-like protein 1 (*ATG16L1*) contributes to CD. In mice, defective autophagy stimulates Rac1 to restrain dendritic cells (DCs) migration [19] and inhibits RhoA activity to affect IECs migration to restore the intestinal mucosa [20].

#### NCF2

The neutrophil cytosolic factor 2 (*NCF2*) gene is considered a very early onset IBD-specific susceptibility gene. A missense variant of this gene causes abnormal binding of its p67^phox^ product to Rac2, resulting in a damaged function of oxidase and thus contributing to the development of IBD [8].

#### TTC7A

Lemoine et al. [21] identified biallelic missense mutations in tetratricopeptide repeat domain 7 A (*TTC7A*) that inappropriately activated RhoA signaling in IBD patients with an immune deficiency.

#### TAGAP

A study showed that T cell activation of the Rho GTPase-activating protein (*TAGAP*) SNP rs212388 negatively correlated with the severity of anal disease in CD, and hypothesized that the protective effect may be attributed to impaired Rho GTPase inactivation [22]. This supposition requires more studies to authenticate.

## Intestinal microbiota

Studies have shown for decades that the intestinal microbiota is essential to IBD development [23]. Intestinal homeostasis partly depends on the mutual influence of intestinal microbiota and the immune system [24] and Rho GTPases are involved in the interaction. For example, segmented filamentous bacteria (SFP), which are commensal gut bacteria in several animal species, induce the differentiation of T helper 17 (Th17) cells in the mouse intestine, which requires Cdc42-dependent endocytosis [25].

Compared to normal person, IBD patients intestinal microbiota communities are markedly different, with decreased abundance of Firmicutes and *Saccharomyces cerevisiae* and increased abundance of Proteobacteria (especially Enterobacteriaceae) and *Candida albicans* [26, 27]. Additionally, the microorganisms expanded in IBD patients can experimentally induce colitis, whereas those contracted are able to ameliorate it [28]. *Escherichia coli*, especially adherent-invasive *E. coli*, participate in IBD, and adherent-invasive *E. coli* strains share similarities with extraintestinal pathogenic *E*. coli such as uropathogenic *E. coli* [29]. Cytotoxic necrotizing factor 1, a protein produced by uropathogenic *E. coli*, modifies Rac2 to restrain it in an active state. Active Rac2 then induces receptor-interacting proteins 1 and 2 (RIP1 and RIP2) to trigger nuclear factor-kappa B (NF-κB) and interferon regulatory factor (IRF) pathways, driving a protective immune response in mammalian cells [30]. Although the connection between enteropathogenic and enterohemorrhagic *E. coli* and IBD has seldom been studied, it has been disclosed that their pathogenesis involves the activation of downstream substrates Cdc42, Rac1, and RhoA as RhoGEFs [31]. Interestingly, with a similar mechanism, *C. rodentium* infects colon cells, inducing IBD-linked inflammation in mice [32]. Moreover, *Salmonella* spp. (family Enterobacteriaceae) produce the pro-inflammatory protein SopE to activate Rac1 and Cdc42, subsequently inducing nucleotide oligomerization domain-containing protein 1 (NOD1)/RIP2/NF-κB signaling, causing an inflammatory response in host cells [33, 34]. Taken together, these studies suggest that Rho GTPases participate in inflammation induced by expanded Enterobacteriaceae in the gut mucosa, thus contributing to the onset of IBD. Additionally, *Clostridium difficile* influences the deterioration and recurrence of IBD [35] probably because its enterotoxins inactivate Rho, Rac, and Cdc42 [ref. [36]]; however, whether this bacterium is associated with IBD development remains unknown. Accordingly, the role and mechanism of action of aforementioned bacteria in IBD require further research.

## Immune system

### The innate immune system

The innate immune system includes non-classical (intestinal epithelial barrier) and classical factors (DCs, macrophages, monocytes, and neutrophils). Dysregulation of these protective components by Rho GTPases is associated with IBD [2, 37] (Table 2).

**Table 2:** Effects of Rho GTPases in the classical innate immune system in IBD.

Immune component	Rho GTPases	Downstream mechanism	Effect	Reference
Intestinal epithelial barrier	Cdc42 Rac RhoA	F-actin	Cdc42, Rac and Rho inactivation results in cell filamentous actin degradation and alter TJ	[36]
		F-actin, occludin, ZO-1, JAM-1	Cdc42, Rac and Rho overactivation results in TJ proteins dysregulation and alter TJ	[41]
	RhoA	RhoA/ROCK/pMLC	RhoA activates ROCK1 and then phosphorylate MLC to alter TJ	[42]
	Cdc42 RhoB	/	Cdc42 and RhoB expression can be upregulated and downregulated by miRNA-21, respectively Cdc42 upregulation and RhoB downregulation increase intestinal permeability and IECs apoptosis	[43, 44]
	RhoA	/	RhoA prenylation decides its membrane localization and activation Cytosolic RhoA accumulation damages IECs cytoskeleton rearrangement	[13]
	RhoA	RhoA/ROCK/pMLC/myosin II	RhoA stimulates ROCK and then phosphorylate MLC to activate myosin II to endocytose TJ proteins in IECs	[46]
	Rac1	Rac1/JNK/caspase-9	Rac1 subsequently activates JNK and caspase-9 to induce apoptosis of IECs	[47]
	RhoA	/	RhoA inhibition in IECs contributes to the apoptosis event	[48, 49]
	RhoA	/	Active RhoA reduction inhibits IECs migration	[20]
	Rac1	/	Rac1 activation enhances migration and cytoskeleton rearrangement in IECs	[50]
	Cdc42	F-actin	Cdc42 expression is enhanced by RNA-binding proteins HuR Decreased Cdc42 interrupts F-actin activation and inhibits IECs migration	[51]
	RhoA	RhoA/ROCK/MLC2, F-actin	RhoA activates downstream effector ROCK and then MLC2 and F-actin to promote IECs migration	[54]
Dendritic cell Macrophage Neutrophil	Cdc42 Rac	/	Cdc42 and Rac regulate DCs endocytosis	[59, 60]
	Cdc42 Rac1	WASp/Arp2/3 complex, PAK/LIMK, MLC, PIP5K/PIP2	Cdc42 recruits WASp to form Arp2/3 complex while Rac1 stimulates PAK/LIMK, MLC and PIP5K/PIP2 signaling to get involved in FcγR-mediated phagocytosis in macrophage	[61]
	Rac2 RhoG	/	Rac2 and RhoG positively regulate FcγR-mediated macrophage phagocytosis	[63]
	RhoC	formin protein mDia1	RhoC activates downstream effector mDia1 to enhance FcγR-mediated macrophage phagocytosis	[64]
	Rac1	/	Rac1 activation promotes TLR4-mediated phagocytosis in macrophage	[65]
	RhoA RhoG	/	RhoA and RhoG are required in CR3-mediated macrophage phagocytosis	[66]
	Cdc42 Rac1 RhoG RhoA	/	Cdc42, Rac1 and RhoG are positive regulators while RhoA is a negativeregulator of integrin-mediated macrophage phagocytosis	[67, 68]
	Cdc42 Rac RhoA	WASp/Arp2/3 complex, RhoA/ROCK, F-actin	Cdc42 and Rac recruit WASp to form Arp2/3 complex while RhoA activates ROCK and F-actin to improve cell migration	[69, 70, 75]
	Rho	ERM protein	Rho stimulates downstream effector ERM protein to promote phagosome maturation	[71]
	Rac1 Rac2	/	Rac1 and Rac2 are essential in the formation and activation of NADPH complex and then promote phagocyte microbicidal function	[8, 72, 73, 78, 79]
	Rac1	/	Rac1 deficiency results in decreased recruitment and accumulation of neutrophils in gut	[10]

*ZO-1* zonula occludens-1, *JAM-1* junction adhesion molecule-1, *pMLC* phosphorylated myosin light chain, *IECs* intestinal epithelial cells, *DCs* dendritic cells, *WASp* Wiskott-Aldrich syndrome protein, *Arp2/3* actin-related protein 2/3, *PAK* p21-Activated kinase, *LIMK* LIM kinase, *PIP5K* phosphatidylinositol-4-phosphate 5-kinase, *PIP2* phosphatidylinositol 4,5-bisphosphate, *TLR* toll-like receptor, *ERM protein* ezrin-radixin-moesin protein, *PI3K* phosphatidylinositol-3 kinase.

#### Intestinal epithelial barrier

Dysregulation of the intestinal epithelial barrier, including abnormal mucous layer [38], damaged structures and functions of the IECs [36], and decreased expression of antimicrobial peptides [39, 40], have been reported in IBD.

Increased TJs permeability, altered cytoskeletal rearrangement, and abnormal cell death in IECs are strongly associated with IBD [13] and Rho GTPases influence IECs through all these aspects. Interestingly, both the overactivation and inactivation of Rho GTPases damage TJs by directly affecting TJ proteins and indirectly affecting the F-actin cytoskeleton intimately associated with TJs [36, 41]. Reduction of cortactin, an actin-binding protein reduced in IBD patients, upregulates RhoA/Rho-associated protein kinase (ROCK) signaling to phosphorylate myosin light chain, which contracts actomyosin leading to altered TJs and increased epithelial permeability [42]. As mentioned above, MYO9B deficiency probably results in the abnormal activation of Rho to interrupt the formation of TJs [16]. Reduced RhoB levels are also associated with impaired TJs that contribute to UC [43] and increased RhoB expression combined with decreased Cdc42 expression ameliorates dextran sulfate sodium (DSS)-induced increased intestinal permeability and IEC apoptosis [44]. Impaired RhoA prenylation and signaling in IECs found in IBD patients drive altered cytoskeletal rearrangements [13]. The cytokines interferon-gamma (IFN-γ) and tumor necrosis factor-alpha (TNF-α) are increased in IBD patients [45], inducing TJ proteins endocytosis and IECs apoptosis through the RhoA/ROCK pathway and the activation of Rac1 triggering the c-Jun N-terminal kinase (JNK) pathway, respectively [46, 47]. *Clostridium difficile* toxins induce IEC apoptosis in a Rho GTPase-dependent manner [48, 49]. Defective mucosal healing is also a characteristic of IBD. RhoA deficiency in IECs is related to CD, with defective IECs migration and intestinal injuries repairment [20], while Rac1 inhibition suppresses IECs migration and inhibits the recovery of colonic wounds in DSS-induced mice [50]. Cdc42 also contributed to mucosal healing in DSS-treated mice [51].

Compared to UC, reduced expression of inducible β- and α-defensins secreted by Paneth cells has been observed in patients with colonic CD [39, 40]. Studies also identify that α-defensins inhibit *Clostridium difficile* toxins by protecting Rac1 from glucosylating [52, 53], while inducible β-defensins improve intestinal wound healing in mice by activating RhoA/ROCK signaling [54].

#### DCs and macrophages

Dendritic cells and macrophages play both similar and different roles in maintaining intestinal homeostasis and inducing an immune response. DCs mainly function as antigen-presenting cells (APCs), as they uptake and present antigens to regulate immune responses, and participate in lymphocyte gut homing [55]. Although macrophages are also considered APCs, their major functions are engulfing and clearing pathogens and apoptotic cells to resolve inflammation [56]. The onset of IBD can be partly attributed to DCs and macrophages incorrect recognition of commensal microorganisms, their abnormal activation and migration, and the subsequent imbalance between immune tolerance and activation [55, 57]. Impaired clearance of microorganisms and apoptotic cells and inflammatory resolution have also reported at the onset of IBD [58].

Endocytosis is associated with antigen uptake and engulfing, which have been shown to depend on Cdc42 and Rac regulation in DCs [59, 60]. In macrophages, this process varies according to the receptor. Cdc42 and Rac1 are recruited to the plasma membrane where Fc gamma receptor (FcγR) is clustered, which then recruits Wiskott-Aldrich syndrome protein to activate the actin-related protein 2/3 complex. The latter activates p21-activated kinase (PAK) and phosphatidylinositol-4-phosphate 5-kinase to regulate FcγR-mediated endocytosis in macrophages [61, 62]. Rac2, RhoG, and RhoC may also participate in this process [63, 64]. Studies have demonstrated that Rac1 promotes toll-like receptor (TLR) 4-mediated bacterial phagocytosis [65] while RhoA and RhoG are required for complement receptor 3-mediated phagocytosis [66]. Furthermore, apoptotic cell engulfing mediated by integrin is enhanced by Rac1, Cdc42, and RhoG, and inhibited by RhoA [67, 68]. DCs require Rac, Cdc42, and Rho involvement to accomplish migration and antigen presentation; the former requires the dynamic regulation and cooperation of Rho GTPases. Rac1/2 and Cdc42 are indispensable in forming lamellipodia and filopodia at the leading edge, respectively, while RhoA controls contractility at the cell rear and migration [69, 70]. In macrophages, the maturation of phagosomes is promoted by Rho/ezrin-radixin-moesin (ERM) protein [71] and macrophages then pretend to exert a microbicidal function. Rac2 is an essential component of the NADPH complex [8] but Rac1 has also been shown to be involved. After phagocytosis, Rac1 is activated and recruited to the plasma membrane, causing the NADPH complex activation and reactive oxygen species generation to remove microorganisms[72, 73]. Furthermore, macrophage complement is a process that includes monocyte adhesion, migration, and differentiation. RhoA and Cdc42 are required for monocyte adhesion, whereas migration requires Rac, RhoA, and Cdc42 participation [74]. The migration mechanism is similar to that aforementioned. Another study found that RhoA functioned in both the front and back of cells, contributing to leading edge construction and governing turning while stimulating actomyosin contractility, respectively [75].

In brief, these studies imply that Rho GTPases may contribute to abnormal DCs and macrophage functions induce IBD pathogenesis; however, direct evidence is lacking to confirm the connection between them.

#### Neutrophils

Abnormal activation, recruitment, and function of neutrophils have been identified in IBD pathogenesis [76, 77]. In neutrophils, Rho GTPases play roles similar to that in DCs and macrophages, i.e., they regulate migration, phagocytosis, and microbe clearing [78, 79], in addition to the degranulation process [80]. A few findings directly demonstrate that dysfunction of Rho GTPases in neutrophils contributes to IBD. Rac1 deficiency in mouse neutrophils ameliorated DSS-induced colitis, probably by inhibiting neutrophil recruitment and migration to the inflammation site [10]. Failure of Rac2 to bind the NADPH complex in neutrophils results in susceptibility to CD [8].

## The adaptive immune system

The adaptive immune system includes T cell-induced cellular immunity and B cell-induced humoral immunity, and the involvement of the former in IBD has been extensively explored [2]. Rho GTPases are crucial for regulating lymphocyte biology [81].

### T cells

**Fig. 2:**
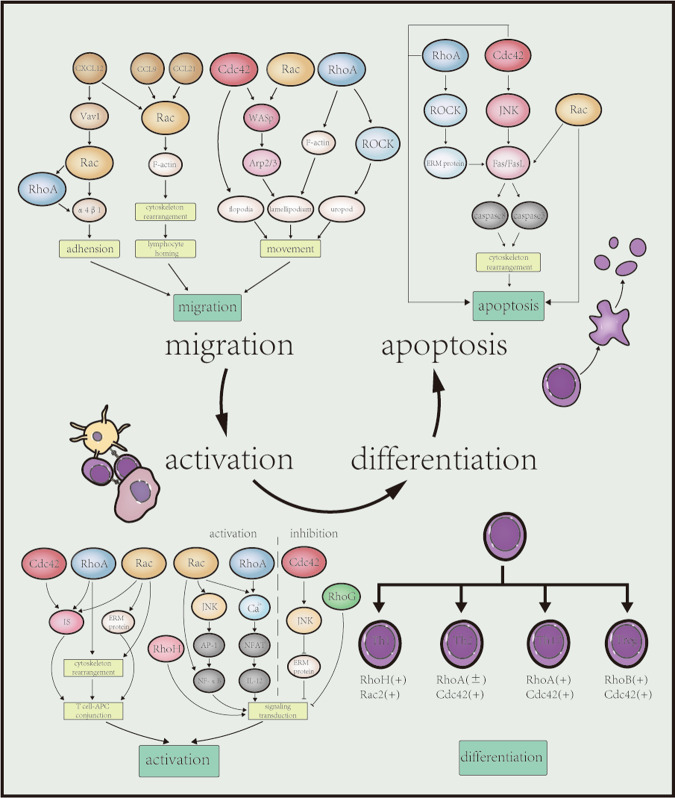
Role of Rho GTPases in the adaptive immune system. Abnormal T cells activation, accumulation, differentiation of different T cells subsets and inappropriate apoptosis are linked to IBD immunopathogenesis. Rho GTPases are involved in T cells basic functions. Migration of T cell initiates with enhanced adhension mediated by Rac and RhoA, the former also participates in lymphocyte homing. Cdc42, Rac and RhoA regulate T cell movement with different mechanisms. Effective T cell activation requires stable conjunction between T cell and APC and the signaling transduction. RhoA and Rac contribute to both the process while Cdc42 enhances the former process and inhibits the latter. RhoH and RhoG are also involved in signaling transduction, mainly exerting a positive and negative function, respectively. Rho GTPases also help lymphocytes differentiate into diverse effector or regulatory T cell subsets to participate in inflammatory and anti-inflammatory processes. Cdc42, Rac and RhoA are involved in the Fas/FasL induced apoptosis of T cell, both in the induction and late stage. WASp Wiskott-Aldrich syndrome protein, Arp2/3 actin-related protein 2/3, IS immunological synapse, ERM protein ezrin-radixin-moesin protein, JNK c-jun N-terminal kinase, AP-1 activate protein-1, NFAT nuclear factor of activated T cells, NF-κB nuclear factor of kappa B, IL-12 interluekin-12.

Abnormal T cell activation, accumulation, differentiation and inappropriate apoptosis are considered IBD immunopathogenesis [2]. Rho GTPases are crucial in adaptive immune response because of their wide control of T cell basic functions (Fig. 2).

#### Migration

Migration of T cells is a sophisticated process. The mechanism of this event is similar, requiring cooperation between Rac, Cdc42, and RhoA [75, 82]. In addition, there is accumulating evidence that Rho GTPases contribute to T cell recruitment. C-X-C motif chemokine ligand 12 (CXCL12) stimulates the Rac1 guanine nucleotide exchange factor (Vav1)/Rac1 pathway to upregulate integrin α4β1 expression to promote T cell adhesion [83], a process that might be RhoA-dependent [84]. Without Rac2, T cells show defective response to CXCL12 and chemokine ligands 19 and 21 and are less efficient at homing to lymph nodes [85]. Once T cells are effectively activated, Rac and RhoA start to inhibit migration. The function of Rac here depends at least partly on the dephosphorylation of ERM proteins [82, 83]. RhoH also negatively regulates T-cell migration [86]. In IBD patients, T cells isolated from gut tissues showed decreased expression of *Pggt1b*, which led to dysfunction of RhoA in IECs and T cells in mice, promoting CD4 + T cell accumulating at the intestine and aggravating colitis [13, 18].

#### Activation

The interaction between T cells and APCs, an essential process for triggering T cell activity, is induced by T cell antigen receptor (TCR) and co-stimulatory molecules, followed by an immunological synapse formation, where active Rac1, Cdc42, and RhoA are observed [87]. Additionally, Rac enhances the connection between T cells and APCs by participating in the dephosphorylation of ERM proteins [88], while inactive Cdc42, Rac, and RhoA impair the activity of co-stimulatory molecules [89, 90]. The upcoming signal transduction process also requires Rho GTPases involvement. The activation of JNKs and transcription factors activator protein-1, nuclear factor of activated T cells, and NF-κB is associated with Rac1[refs. [91, 92]]. Rac1 also influences Ca2+ influx into T cells [93], an event involved in the maintenance of this interaction [94] and in cytoskeleton reorganization during signal transduction [94, 95]. The latter probably depends on the phosphoinositide 3 kinase (PI3K)/Vav1/Rac or Rho pathway [95]. Inhibition of RhoA leads to damaged Ca2+ influx [96] and RhoA is involved in T cell activation through the regulation of mitochondrial function [97]. Cdc42 deficiency results in the overactivation of murine T cells [98]. Moreover, RhoG and RhoH have been reported to regulate the TCR signaling pathway, mainly as the suppressor and enhancer, respectively [86, 99].

#### Differentiation

After activation, T cells differentiate into various subtypes that perform different functions. Generally, CD was considered a Th1 condition and UC an atypical Th2 condition. With the observation of other subsets engagement, such as Th17 and regulatory T cells (Tregs), this view has changed [2]. As mentioned above, Cdc42 is required for SFB-induced Th17 differentiation in mice [25]. Deficiency of Cdc42 promotes the differentiation and pathogenicity of Th17 and disrupts Tregs differentiation and stability, aggravating DSS-induced colitis [100]. Deficiency of Cdc42 in mice also interrupts Th2 differentiation [101]. RhoA induces Th2 and Th17 differentiation, and the former occurs partly via defective T cell metabolism [97, 102]. However, another study suggested that decreased activation of RhoA, because of its abnormal membrane location, contributes to a shift from Th1- to Th2-modulated immunity through the inhibition of p38 [103]. In Th1 cells, Rac2 triggers the NF-κB and p38 signaling pathways to upregulate IFN-γ expression [104]. As for RhoH, a study found a remarkably higher differentiation level of Th1 than Th2 [105]. Additionally, decreased RhoB activity promotes Tregs differentiation [106].

#### Apoptosis

Cdc42 activates JNK and upregulates Fas and Fas ligand (FasL) expressions to induce T cell apoptosis in secondary lymphoid organs [107]. Cdc42 and Rac are associated with both the induction and late stages of Fas/FasL-induced apoptosis but do not directly participate in it [108], and activated Rho/ROCK signaling is also involved in the process by phosphorylating ERM proteins [109]. Interestingly, decreased RhoA activation has been reported to contribute to T-cell apoptosis [110]. Activated Rac1 with Vav1 restrains the NF-κB and signal transducer and activator of transcription 3 (STAT3) signaling pathways to induce T cell apoptosis to alleviate clinical symptoms in IBD patients [111, 112].

### Cytokines

Cytokines include interleukins (ILs), colony stimulating factors (CSFs), IFNs, TNFs, transforming growth factor-β family members, growth factors, and chemokines. The role of these molecules has been gradually explored in IBD [113]. Cdc42 has been shown to downregulate IL-4, IL-10, TNF-α, and IFN-γ expression both in the colon tissues and serum of 2,4,6-trinitrobenzenesulfonic acid (TNBS)-induced mouse models to regulate inflammation [114]. Conversely, TNF-α downregulated Cdc42, while CdC42 overexpression reversed the intestinal epithelial barrier damage due to TNF-α in Caco2 cells [115]. RhoA/ROCK pathway activation stimulates the production of TNF-α and IL-1β, and there is a positive correlation between RhoA and TNF-α in the intestinal inflammatory tissues of CD patients [116]. Inhibition of the RhoA/ROCK pathway also disrupted TGF-β-induced fibrosis in human [117]. RhoA dysfunction in T cells promoted IL-1β, IL-17A, TNF-α, and IFN-γ expression in mice developing spontaneous colitis [18] and blocking RhoA activation reduced IL-2 production [96]. In addition, Horowitz et al. [118] identified that TNF-α/lipopolysaccharide could activate the RhoA/ROCK pathway and contribute to IBD. Rac1 activity is upregulated by TGF-β in Caco2 cells to promote migration and epithelial restitution [119]. Rac1 can also be activated by CSF, IL-6, IL-8, and TNF-α [120]. Inhibition of Rac1 decreases IL-8 level, thus regulating immunocyte migration to the intestinal tract to maintain local remission [120, 121]. Rac1 also suppresses tryptophan-induced reduction of TNF-α, which damages intestinal barrier [122]. In DSS-treated mice, Rac1 deficiency in macrophages and neutrophils results in lower levels of IL-1β, IL-12, and TNF-α [10] whereas a Rac1 inhibitor blocks engulfment and cell motility protein 1-mediated downregulation of IL-1α, IL-6, IL-12B, IL33, TNF-α, and TGF-β to disrupt its protective function in DSS-induced mice [50]. Furthermore, Rac2 is associated with IFN-γ and IL-17A production, suggesting that impaired Rac2 function leads to IBD development [7, 104], and Rac2 inhibition can be used to treat IBD patients [112].

## Environment

Smoking has been considered as a risk factor for CD but a protective factor for UC [123, 124]. Nicotine, a component of cigarettes, activates α7 nicotinic acetylcholine receptors (α7nAChRs) on plasmacytoid DCs to disrupt their migration into colonic mucosa. This protects cells from UC via activated JAK2-STAT3 signaling and the subsequent degradation of Rac1 induced by non-apoptotic caspase-3 [ref. [124]]. However, whether the mechanism of smoking-induced CD involves Rho GTPases remains unclear. The connections between Rho GTPases and other environmental factors contributing to the onset of IBD [124] have been scarcely explored. A study identified that the activation of vitamin D receptor via the RhoA/Rho kinase pathway partly inhibited the inflammatory response in benign prostatic hyperplasia [125] and myocardial ischemia-reperfusion injury [126]. In addition, vitamin D deficiency is associated with IBD [123], suggesting a possible involvement of Rho GTPases in vitamin D-deficiency-induced IBD. Therefore, there is an urgent need to investigate this association.

## Novel pathogenic components

In addition to the aforementioned classical pathogenic factors, several novel components in the pathogenesis of IBD, such as pattern recognition receptors (PRRs), non-coding RNAs (ncRNAs), angiogenesis, and fatty acids, have gradually drawn researchers’ attention [2, 127, 128]. Rho GTPases are also participants in these pathogenic processes.

### PRRs

Pattern recognition receptors, including receptor families such as TLRs and NOD-like receptors (NLRs), recognize pathogen-associated molecular patterns and damage-associated molecular patterns to initiate an immune response and regulate other events [129].

#### TLRs

TLRs are transmembrane proteins that participate in innate immunity. Upon activation, TLRs trigger downstream signaling cascades to activate NF-κB, which results in pro-inflammatory mediators secretion to regulate adaptive immunity. The expression and function of TLRs are irregular in IBD patients, suggesting that TLRs are both positively and negatively associated with IBD [130]. Rho GTPases take part in TLR-mediated immune response. In monocytes, Rac1 and RhoA are vital in TLR2-induced NF-κB activation via a Rac1/PI3K/protein kinase B pathway [131] and at least partially through atypical protein kinase C [132]. Rac1 also participates in the TLR1-mediated signaling pathway in epithelial cells [133]. Rac, RhoA, and Cdc42 are all activated after TLR4 is stimulated to promote uptake by macrophages [134, 135]. TLR7/9-mediated production of type I IFN requires Rac involvement in DCs [136]. In macrophages, following TLR activation, Rac acts as a positive regulator of phagocytosis and oxidative burst because of the downregulation of TNF-α-induced protein 8-like 2 [ref. [137]] whereas RhoB enhances the expression of pro-inflammatory mediators by binding to major histocompatibility complex class II [138]. In addition, Cdc42 is involved in TLR-activated DCs-T cells interactions [139].

#### NLRs

NLRs are a group of cytoplasmic proteins whose downstream signaling pathways mainly result in NF-κB and caspase-1 activation [140]. Mutations and dysfunction of NLRs are associated with IBD [140]. Rho GTPases are implicated in the functions exerted by NLRs. After activation, NOD1/2 starts to form a complex (the nodosome) containing Rho GTPases, which is necessary for the activation of the complex itself and NF-κB eventually [34, 141]. Interestingly, Rac1 negatively regulates the NOD2-mediated NF-κB activation and IL-8 induction [142]. In mouse macrophages, the NLR family pyrin domain containing protein 3 (NLRP3) inflammasome senses Rac2 activation and is then phosphorylated by p21-activated kinase 1/2, the substrate of Rac2, to induce the secretion of IL-1β, which contributes to microorganisms clearance [143]. Rho A is also a positive regulator of NLRP3 in Caco2 cells [144]. Rac1 and RhoB are associated with the NLRP3 inflammasome as well [145, 146].

### ncRNAs

MicroRNAs (miRNAs, miR) are among the most extensively studied ncRNAs, as they can silence gene expression by regulating messenger RNAs (mRNAs) translation or degradation [147]. Associations between miRNAs and IBD have been proposed [148–150]. For instance, MiR-15a inhibits Cdc42 expression in pediatric IBD patients, resulting in a damaged intestinal epithelial barrier [116]. MiR-21 targeting and regulation of RhoB expression, which is upregulated in UC, disrupts the intestinal epithelial barrier by damaging TJs [43]. Cdc42 is also regulated by miR-21 in the intestinal epithelial barrier regulation [44]. MiR-31-3p alleviates colitis in IBD mouse models, possibly by targeting and negatively regulating RhoA expression, thus reducing the levels of several cytokines in colonic epithelial cells [151]. Circular RNAs (circRNAs), another class of ncRNAs with covalently closed circles, interact with miRNAs and proteins to perform their functions [152]. The circRNA homeodomain-interacting protein kinase 3 (circHIPK3) was recently identified as a sustainer of intestinal epithelium homeostasis and was reported to be decreased in patients with IBD [153]. CircHIPK3 is, at least partially, directly connected to and thus inhibits miR-29b availability to enhance the expression of Rac1 and Cdc42, promoting epithelial recovery in wounded IECs as well as intestinal epithelium renewal in mice [153].

### Angiogenesis

One crucial event in chronic inflammatory diseases is angiogenesis, a process enhanced in the intestinal inflamed site of IBD patients, and vascular endothelial growth factor (VEGF) and IL-8 seem to act as inducers of this process [154]. Rho GTPases are implicated in this process, mainly in controlling cell migration [155]. Activated Rac1, Cdc42, and Rho, probably via steroid receptor coactivator/focal adhesion kinase signaling, participate in VEGF-induced angiogenesis [156, 157]. Inhibition of Rac1 and RhoA cause defected IL-8-induced endothelial cell migration [158]. Nevertheless, future studies should address the direct evidence of Rho GTPases as participants in angiogenesis, contributing to disclose the molecular development of IBD through more mechanistic studies.

### Fatty acids

Whether the intake of fatty acids is beneficial or detrimental in IBD is still controversial, as several studies have achieved different conclusions [127, 128]. Turk et al. [159] found that n-3 polyunsaturated fatty acids damaged epidermal growth factor receptor-mediated Rac1 and Cdc42 activation to delay intestinal wound healing in DSS-treated mice.

### Clinical Prospect of Rho GTPases

Emerging studies have shown that Rho GTPases act as therapeutic targets in many diseases, including cancer [160], cardiovascular disease [161], and Alzheimer’s disease [162]. As Rho GTPases have a great influence on IBD pathogenesis, their role in IBD treatment has been gradually explored (Table 3).

**Table 3:** Rho GTPases associated therapies in IBD.

Rho GTPases	Drugs	Models	Effect	Reference
Cdc42	AMP-18	Human colonic adenocarcinoma Caco2/bbe (C2) cells	Cdc42 activation stimulates TJ formation	[165]
	Infliximab	UC patient peripheral blood mononuclear cells	Elevated Cdc42 is positively associated with infliximab treatment response	[170]
Rac1	Nsc23766	Human monocytes	Rac1 inhibition enhances phagocyte phagocytosis and microbe clearance	[120]
	Thiopurines	Human DCs	Rac1 inhibition corrects defect DCs migration	[19]
	Thiopurines	Human monocytes	Rac1 inhibition enhances monocytes phagocytosis	[120]
	Thiopurines	IBD patients T cells	Rac1 inhibition induces the apoptosis of T cells	[111]
	Thiopurines	Human T cells	Rac1 inhibition reduces effective T cell-induced immune response	[112]
	Thiopurines	Caco-2 cells	Rac1 inhibition represses macrophages and nuetrophils migration to the intestine	[121]
	Thiopurines	CD patients NK cells	Rac1 inhibition induces the apoptosis of NK cells	[175]
RhoA/ROCK	C3 exoenzyme	Human tissues and isolation of endothelial cells	RhoA/ROCK inhibition corrects microvascular dysfunction	[118]
	Y-27632	Human tissues and isolation of endothelial cells	RhoA/ROCK inhibition corrects microvascular dysfunction	[118]
	Y-27632	Rats with TNBS-induced colitis	RhoA/ROCK inhibition interrupts leukocyte-endothelial interaction	[116]
	Y-27632	Mice with DSS-induced intestinal inflammation	RhoA/ROCK inhibition alleviates intestinal inflammation	[180]
	Y-27632	CCD-18co cells Human intestinal fibroblasts	RhoA/ROCK inhibition suppresses fibrogenesis in colonic myofibroblasts	[117]
	Oxymatrine	Mice with DSS-induced colitis	RhoA/ROCK inhibition alleviates intestinal inflammation	[181]
	Umbilical cord Placenta-derived mesenchymal stem	Human primary intestinal myofibroblasts	RhoA/ROCK inhibition suppresses fibrogenic activation in HIMFs	[182]
	Hydrolyzed guar gum	Mouse colonic epithelial cells	RhoA/ROCK activation promotes colonic epithelial cell wound recovery	[183]
Cdc42 Rac1 RhoA	β-glucan	IEC-6 cells	Cdc42, Rac1 and RhoA activation enhances intestinal epithelial cell proliferation and migration	[184]

*AMP-18* Antrum Mucosal Protein-18, *TJ* tight junction, *DCs* dendritic cells, *NK cells* nature killer cells, *TNBS* trinitro-benzene-sulfonic acid, *DSS* dextran sodium sulfate, *HIMFs* human primary intestinal myofibroblasts.

#### Cdc42

Antrum mucosal protein 18 (AMP-18), which is produced by antral mucosal epithelial cells [163], positively regulates intestinal epithelial TJs stability and mucosa recovery [164]. Cdc42 is activated after treating with AMP-18 and contributes to the formation of TJs [165]. The anti-TNF antibody infliximab, for example, has been proven to be effective in the induction and maintenance of clinical remission in IBD patients [166–169]. Recently, a study found that Cdc42 is upregulated in patients with UC in response to infliximab [170], but the mechanism underlying this upregulation still requires further studies.

#### Rac1

Nsc23766 is a Rac1-specific inhibitor that targets Rac1 binding and activation by RhoGEFs [171]. By moderately blocking Rac1 activation, Nsc23766 enhances phagocyte function in CD patients regarding the ability to clear the microorganisms [120]. Thiopurines is an effective drug to treat IBD [172] and 6-thioguanine triphosphate, one of the main metabolic products that exerts such function, directly binds to Rac1 to inhibit its activation [173]. A clinical trail found that IBD patients response to thiopurine therapy had a lower Rac1 level or activity [174]. Inactivated Rac1 has distinct effects on various cells. Inactivated Rac1 improves the defective migration of DCs in CD patients with the ATG16L1 mutation [19]. In monocytes, when Rac1 activity is inhibited within a certain range, phagocytic function is enhanced [120] but in T cells this change results in a reduced immune response and increased T cell apoptosis due to the suppression of T cell-APC conjugation and impaired NF-κB and STAT-3 signaling, respectively [111, 112]. In Caco2 cells, the migration of macrophages and neutrophils to the gut is suppressed by the downregulation of IL-8 expression [121]. In addition, NK cells, whose role in IBD has been recently discovered, undergo enhanced induction of apoptosis mediated by caspase-9 [ref. [175]]. Overall, thiopurines seems to function as Rac1 inhibitors in IBD treatment. Another kind of clinical drug statins are also demonstrated to suppress Rac1 and RhoA [176] and alleviate CD patients’ inflammation [177]. Moreover, a few clinical trials, working on estimating the efficacy and safety of statins in UC patients, are about to start. However, the activation of Rac1 also shows a possible benefit in IBD patients because active Rac1 improves IECs migration and mucosal healing [50, 119].

#### RhoA

Inhibitors of RhoA and its downstream effector ROCK are C3 exoenzyme [178] and Y-27632 [179], respectively. Both C3 exoenzyme and Y-27632 disrupt arginase expression in human intestinal microvascular endothelial cells, which likely promotes intestinal microvascular endothelial function [118]. Y-27632 has been widely used in previous studies. By interrupting the RhoA/ROCK pathway, Y-27632 has been shown to improve TNBS-induced colitis in rat by inhibiting NF-κB activation [116], alleviating DSS-induced intestinal inflammation by regulating cell function and cytokine secretion [180], and repress fibrogenesis by blocking RhoA/ROCK/MYLK/SRF signaling in colonic myofibroblasts, representing a new therapy for intestinal fibrosis in IBD patients [117]. Furthermore, several substances have shown therapeutic potential by affecting RhoA/ROCK signaling. Oxymatrine, a Chinese herb extract [181], and umbilical cord- and placenta-derived mesenchymal stem cells [182] suppress this pathway and thus alleviate intestinal inflammation in DSS-treated mice [181] and inhibit intestinal fibrosis [182], respectively. However, blockade of signaling can also be detrimental. Partially hydrolyzed guar gum, a product of guar gum, was observed to ameliorate colitis in mice [183]. Recently, a study has found that it improves wound healing in colonic epithelial cells, which is inhibited by Y-27632 [ref. [184]]. Thus, RhoA/ROCK signaling pathway regulation might provide a new insight into IBD treatment. β-glucan, which upregulates the expression of Cdc42, Rac1, and RhoA, enhances cell migration and proliferation, and ultimately promotes intestinal mucosal wound recovery, also represents a therapeutic potential for IBD via mucosal healing [185].

Although so far Rho GTPases targeted drugs haven’t been widely applied and tested in clinic, many studies have proven that several IBD therapies influence the signal transduction pathway of Rho GTPases and their regulators are effective in improving intestinal inflammation in animal experiments. Additionally, inhibition of Rho GTPases are used in other immunological disease treatments, such as rheumatoid arthritis[186] and asthma [187]. Therefore, regulation of Rho GTPases might be clinically useful in treating IBD but still waits for further researches and larger clinical trials.

## Conclusions

Accumulating evidence has revealed that Rho GTPases are associated with IBD pathogenesis. According to the aforementioned studies, whether Rho GTPases have a beneficial or detrimental effect on IBD development remains unclear. Studies of Rho GTPases in IBD conducted so far have been mostly based on static conditions using cells and/or animal models, and have been limited by the deficiency of clinical samples. As numerous cell events depend on Rho GTPase dynamics, it is difficult to draw a connection between Rho GTPases and the development of IBD.

Nevertheless, therapies targeting Rho GTPases and their regulators are promising. Rho GTPase inhibitors have been widely studied and have been shown to be beneficial in alleviating disease. In addition, the mechanism of thiopurine therapy, one of the most used treatments in the clinical setting, requires Rho GTPase inactivation. However, several studies pointed out that Rho GTPase activation could also be a promising therapy. Together with the fact that extensive basic fundamental events require Rho GTPases involvement, general inhibition of Rho GTPases might not only provide the desired therapeutic effect, but also be detrimental to the individual [188]. Additionally, thiopurine therapy is limited by its long-term treatment and potential side effects. Thus, new drug discovery and fewer adverse reactions are future research directions for therapies targeting Rho GTPases. Furthermore, future studies should also focus on other Rho GTPases that have been implicated in the onset of IBD. In summary, Rho GTPases are associated with the pathogenesis of IBD, and further clinical studies are required to confirm and explore the underlying mechanisms. The Clinical Prospect of Rho GTPases is promising and require further studies on safety improvements and novel drug research development.

## Data Availability

All data included in this review are available upon request by contact with the corresponding author.
